# Open Access as a Revolution: Knowledge Alters Power

**DOI:** 10.2196/16368

**Published:** 2019-12-11

**Authors:** Dave deBronkart

**Affiliations:** 1 e-Patient Dave, LLC Nashua, NH United States

**Keywords:** patient engagement, empowerment, patient empowerment, participatory medicine, open access, patient portals, EMRs, EHRs, Patient-clinician relationship

## Abstract

The slogan “Gimme My Damn Data” has become a hallmark of a patient movement whose goal is to gain access to data in their medical records. Its first conference appearance was ten years ago, in September 2009. In the decade since there have been enormous changes in both the technology and sociology of medicine as well as in their synthesis. As the patient movement has made strides, it has been met with opposition and obstacles. It has also become clear that the availability of Open Access information is just as empowering (or disabling) as access to electronic medical records and device data. Knowledge truly is power, and to withhold knowledge is to disempower patients. This essay lays out many examples of how this shows up as we strive for the best future of care.

## Introduction

As I write these words on September 17, 2019, it has been ten years since I gave my first keynote speech at a medical conference in Toronto, at Medicine 2.0. My opening slide had the date wrong, but this was it: the first time Gimme My Damn Data was spoken on stage [1].

It was ten years into the Journal of Medical Internet Research’s (JMIR) long history, the midpoint between today and its founding. Most of what I wrote in my blog post about that speech [2] is still valid; most importantly, “there’s still no way for me to get all the data out” of my hospital, Boston’s Beth Israel Deaconess. Ironically, they are considered a leader in health information technology. Sociologically they are, as it is the home of OpenNotes; however, that is a social movement, not a technology.

The genesis of my advocacy for data rights is a story of its own: I believed my hospital’s (former) Chief Information Officer (CIO) when he blogged [3] that I could move my medical data into the old Google Health site, and they were wrong. In short, a blog post I wrote about garbage that showed up in Google Health landed on the front page of the Boston Globe. The resulting social media furor led to a speaking invitation from Gunther Eysenbach, Editor-in-Chief of JMIR. He kept asking what title I would use, and in frustration, I told him to “Just call it ‘gimme my damn data,’ because you guys can’t be trusted with it.” The first part stuck.

That speech turned into a movement, a music video by Ross Martin, MD, and his band [4], and an unplanned global speaking career. Along the way, Ross’s wife Kym, a multi-cancer survivor, made it more polite and descriptive by changing it to DaM data: Data About Me. However, health care institutions mostly do not get it yet, so I say that it is time for a revolution.

**Figure 1:**
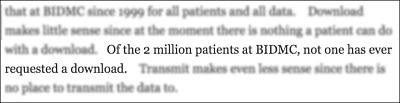
Blurred screen capture with extract "Not one patient".

That former CIO, who knows me personally, has nonetheless blogged that patients do not want their information and that there is nothing they could do with it if they had it [5,6]. He wrote that not a single patient at our hospital has ever tried to download their record (Figure 1), and then we learned he does not have a way to do that [7]. I am fed up with this kind of “leadership,” even though I am not in a health crisis. If I were in an emergency, I would be furious because knowledge and information are power, so to withhold patient data is to disempower the person for whom the profession exists. I will not stand for it anymore. To me, the withholding of information from patients is the same issue as putting general medical information behind paywalls, an issue that favors Open Access journals that freely provide knowledge, a model which JMIR has pioneered.

In this article, I will write briefly about ten significant developments that have concurred with JMIR’s first twenty years, with the developments divided into three categories: technological, sociological, and the convergence of the two. In the technological category, these developments include the arrival of electronic medical records (EMRs) and government mandates, particularly in the United States. The sociological category has to deal with the awakening of the patient movement, which includes developments such as the empowered, engaged, equipped, and enabled patient (e-patient) movement (citing Eysenbach), uncovering what empowerment is, the #PatientsIncluded movement (“Nothing about me without me” scales up), resistance from the establishment, parallels with the women’s movement, and the British Medical Journal’s Patient-Public Partnership. Finally, the convergence between the two involves the awakened and empowered seeking their data, with new technology enabling it, and includes the emerging standard of Fast Health care Interoperability Resources (FHIR).

## Development 1: Health Information Technology Regarding Electronic Medical Records and Mandates

Undeniably the first significant development in the ten years since my speech was the arrival of US $40 billion in subsidies and then penalties that pushed the country toward finally computerizing our medical data. The first electronic medical record systems had arrived in the previous century, but they did not take off until the industry was forced to acknowledge them. I shudder to think what the state of adoption would be today if it were not for that $40 billion.

The usability of these systems is widely considered to be horrible [8]. However, as I previously said on Twitter [9], blaming the government is off target. A few months after my speech in Toronto, while in Washington D.C., I referred to rumors that an EMR vendor Chief Executive Officer (CEO) had said usability would be a system criterion “over my dead body.” The vendor won, but though adoption has been ugly, the situation has progressed to the point where we do have information systems. For the first time, it is possible to move data around, and though we are nowhere near the finish line, we have finally moved off the starting line.

## Development 2: The E-Patient Movement and Participatory Medicine

**Figure 2:**
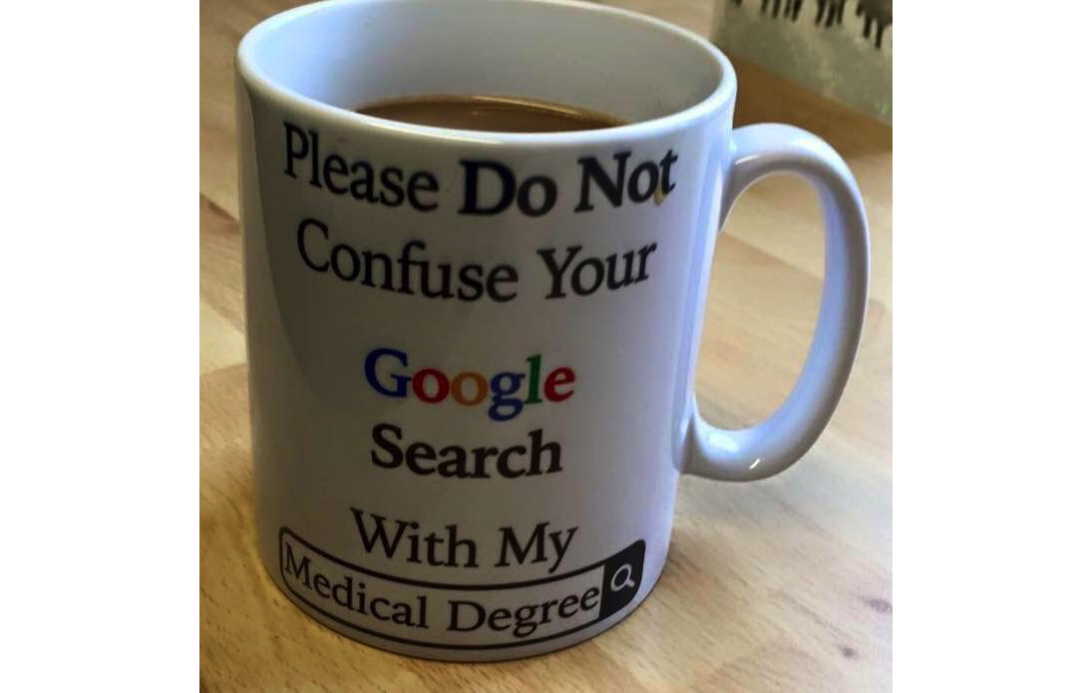
Google mug.

“Doc Tom” Ferguson was a visionary who saw that access to information via the internet would transform what patients could do to contribute to their family’s health and care. His “e-Patient White Paper” [10], published posthumously in 2007, cited Gunther Eysenbach’s remarkable DAERI [11] project, which established that Googling does not lead to a flood of deaths. He found zero deaths in years of seeking horror stories, even when he offered a €50 (US $55.02) bounty. Trying not to view internet research done by patients negatively is a lesson that could still be learned by medical students today, as highlighted by the Google mug meme that has been circulating online (Figure 2) [12].

To my knowledge, Eysenbach and Doc Tom produced the first crack in the armor of the idea that only doctors can know what is worth knowing. All else has stood on their shoulders. The same year of that Medicine 2.0 speech, Ferguson’s founders formed the Society for Participatory Medicine [13].

## Development 3: Understanding What Empowerment Is

One of the potential definitions of the “e” in Ferguson’s e-patient concept that is often cited is empowerment. However, in my first years of conference travels, I found universal uncertainty about what we mean by it. If we are not clear about what empowerment is, how can we even tell whether someone is doing it? Then, at the 2013 World Parkinson Congress in Montreal, Swedish e-patient Sara Riggare told me about a definition she had seen in a speech the previous day. This was a definition that the World Bank has been using since 2002 when they help a developing nation become self-reliant [14]:

The process of enhancing an individual’s or group’s capacity to make purposive choices and to transform those choices into effective actions and outcomes.

Isn’t that precisely what we seek in empowering patients? Increase their capacity to make choices and produce results? Someone who can do that has power, and someone who does not is disempowered. With this straightforward definition, we can evaluate any intervention: does it increase or diminish one’s capacity? I believe that withholding one’s medical data diminishes their ability to manage their medical lives. Access is empowering. I assert that the same applies to medical literature, which can be inaccessible to patients because of paywalls. It is bluntly disempowering to the patient who has the problem because the publisher has a higher priority than directly empowering the sick person and their carers.

I asked Sara which scientist or sociologist had presented this at the conference. She said it was a fellow patient, Fulvio Capitanio, who said it during his presentation. It is important to note that it was not a scientist who was moved to present this as a topic of significance, but a patient.

## Development 4: The Genesis of #PatientsIncluded

In 2013, the Dutch visionary Lucien Engelen posted that he had seen the future and would no longer speak at any health conference, not even for a hefty fee, if it did not actively include patients, on stage or in the audience, which included paying their expenses [15]. He was the first leader I know of who said we could not do health care right (or plan or manage it) if patients are not involved in the process. Today, among other things, this includes patient data management.

As with many social issues, #PatientsIncluded is the latest expression of an earlier principle: Nothing about me without me. This concept started with the disability rights movement in South Africa in the 1980s [16], but they had another potent line about showing up to advocate for yourself: “When someone else speaks for you, you lose” [16]. The women’s suffrage movement found the same in the 1800s when they let some men speak for them in Congress [17]. The men made compromises that the women would not have accepted.

## Development 5: Discovering Parallels With the Women’s Suffrage Movement

Speaking of the women’s movement, I was in the Boston college scene when feminism rolled through, and I have noticed many parallels with the participatory medicine movement. One such favorite parallel of mine is that at dances, we had to shift from “boy leads, girl follows” to a bit more collaboration, just as participatory medicine tells us to share taking the lead. Also, in 1912, some people resisted women’s suffrage by saying, “Most women aren’t asking for it” [18], just as some clinicians today say, “My patients aren’t asking for access.” Seeing the potential for improvement, and enabling it, requires vision. Saying “I don’t see the potential yet” is a favorite way to stay secure in the past. That never improves things.

## Development 6: Resistance

When you are in a revolution, at first, they ignore you. When they start to strike back, it is a sign that those you are fighting against are waking up. I was appalled in 2014 when Dutch visionary Lucien posted about two award-winning commercials (published by the Belgian government) that said, “Don’t google it. Check a reliable source”, as if that was an either-or proposition. e-patients who are using Google to get information are trying to supplement their knowledge, not ignore doctors; we may google it, and we can also check a reliable source. However, in both commercials, the citizen falls for an incorrect website and does not think to ask their clinician [19,20]. Not to be outdone, Israel’s largest health maintenance organization, Clalit, produced a commercial [21] showing a “search victim” rolled into an emergency room. A doctor yells, “Clear!” and slams the patient’s laptop shut as if turning off the computer was as potent as cardioversion.

Savvy e-patients know there is incorrect information on the internet, and they know not to swallow everything they see. In 1999, I found my wife on match.com. Before I found her, I got some suboptimal search results, but I knew enough to check before acting. If you have ever taught a child how to be careful online, you know that when someone is inexperienced, the remedy is not to keep them naïve but instead to teach them how to watch out for themselves. Paternal caring is essential when someone cannot make choices, but it is cruel and foolish when they have grown up.

There is a sad side to getting this wrong. The NHS Trust in Nottingham, England, had to apologize to the family of 19-year-old liver cancer patient Bronte Doyne, who died after her doctors told the family to stop going to Google for information, even though the family had found valid information that the clinicians did not know [22]. Ironically, these physicians had never received training about how valid information can be found online. Physician naïveté about this issue is a legitimate challenge to physician credibility and authority.

In an era of constant and overwhelming change, it is unfair to put all the burden on the health system. Sometimes a patient might discover something the doctors have not seen, and an enlightened doctor will know that and not squander the opportunity. This is not about ignoring doctors. It is about patient participation to help health care achieve its potential.

## Development 7: Discovering Parallels With Radically Rethinking Education

The radical education book “Pedagogy of the Oppressed” [23] discusses how you teach people, under the old model of “teacher knows everything useful; student is an ignorant puppet, a bucket to be filled properly.” The book asserts that this is oppressive. The more “right” approach, it says, is to teach the student to question, to think critically for themselves. Can you sense the parallels with “doctor knows best” medicine and participatory medicine?

The Society for Participatory Medicine’s cofounder Sarah Greene penned a post in 2009 called, “Participatory Medicine as Revolution: Think Critically! Communicate!” [24] She replaced the book’s use of student and teacher with patient and provider:

Patients, having adopted guidelines of their health care provider and internalized his images, [become] fearful of freedom. Freedom would require them to eject this image and replace it with autonomy and responsibility.

Only this year did I connect the dots between this and how information empowers, which in turn enables revolution. When we have limited access to information, we are dependent, whether anyone intended us to be or not.

A note that is relevant to JMIR, in particular, is that information behind a paywall impedes growth and empowerment. Open access to information is empowering because it enables the citizen.

## Development 8: Convergence of Technology and the Social Awakening With the Science of Behavior Change

For years, my mind has itched at the complaint, “My patients aren’t asking for this.” But then I remembered meeting Stanford behaviorist BJ Fogg in 2010, with his model of behavior change: Behavior change is a function of motivation to change, ability to take the requested action, and some trigger (or prompt). Someone might write it out as:

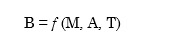



However, he writes it as B=MAT (in later versions prompt replaces trigger). He developed this into a useful 3 × 5 matrix of types of behavior change, which I have rarely heard discussed in health care and never regarding health data. He breaks it up as follows:

- Y-axis: “Dot, span, or path.” Are we talking about doing something once (dot), or for a specific time (span), or permanently (path)?
- The X-axis is a type of change: start something, end something, do more or less of something, or do something they already know how to do (once, or for a while, or permanently).

With this model, if someone complains that patients “don’t do as they’re told,” we are able to ask, “Well, what type of change were you requesting?” Moreover, if they say patients are not asking for something (such as data), we can explore differences between observed groups rather than assuming that all patients are the same. In behavior change as in biology, a more thoughtful diagnostic approach has a better chance of helping patients.

I suspect we will discover a role for Freire’s advice to awaken the “student.” As he said, that will never happen if we think patients are mindless vessels to be filled by somebody else. Could it be that our paradigm of patient is a principal cause of the behaviors people complain about [25]?

## Development 9: Motivation for Action Due to the Business Side of US Health Care

I am not happy to say this, especially since I have come to know many good, committed people who work in this system, but there are many instances of mistreatment in the system:

- “If Grandma Is on the Table, No One Will Blink at the Price”: A Former Drug Company Manager Explains Industry Price-Setting [26]
- “Is curing patients a sustainable business model?” (Investment company Goldman Sachs) [27]
- Stories of hospitals suing their patients to preserve their health at the cost of the patient’s health
  - “‘UVA Has Ruined Us’: Health System Sues Thousands Of Patients, Seizing Paychecks And Claiming Homes” [28]
  - “When Hospitals Sue For Unpaid Bills, It Can Be 'Ruinous' For Patients” [29]
  - “As Patients Struggle With Bills, Hospital Sues Thousands” [30]

We must revolt against this, or the businesspeople who run parts of the health care system shall surely crush us. However, to revolt and strive for autonomy, we need information.

## Development 10: Fast Health Care Interoperability Resources

At this twentieth anniversary of JMIR, and the tenth anniversary of my first speech, there is new hope for autonomy and independence due to a revolution empowered by information. A new software standard for data mobility, FHIR, is maturing. In my personal view, this is the most significant change in these past ten years; the signs are that FHIR might make real what I was trying to do in 2009 with Google Health. I say this in part because a familiar origin story for a patient’s newfound empowerment is their discovery of a significant mistake in their medical information, or even discovering that vital information from one care provider never made it to the next. Sometimes the failure of that data to move to the point of need causes horrific harm.

## Tying it All Together

So much has changed since September 2009, but the business side of health care still says that patients should do as they are told. A few years ago, Judy Faulkner, founder and CEO of EMR vendor Epic, famously argued with former US vice president Joe Biden, saying, “Why do you want your medical records? They’re a thousand pages of which you understand 10.” Nevertheless, Biden replied, “None of your business.” [31] Biden’s motivation trumped her apparent belief that he (and his son’s doctors) could not make use of the asset. Biden had lost his son Beau to brain cancer, and the final efforts to save him were impeded by the inability to move Beau’s data around. So, one can imagine that it would be upsetting to Biden when someone criticizes his desire to help people save their families. There is a video of Biden’s speech that same year at Health Datapalooza, where you can sense the mood of someone who tried to help save his son’s life and was blocked [32].

Just this September, both the American Medical Association and the American Hospital Association cautioned against letting patients have their data [33]. In summary, they said, “something bad might happen” if it leaked. That is paternalism at its finest, or in Fogg’s model, “Ability is zero, so no change is possible—why even try?” Empowered patients might respond: “Let me decide!”

It is not just EMR data, too. Many patients have devices wired into their bodies. These are real medical wires and sensors under their skin, and some of these patients want to see the data their devices are generating. They are empowered, an attitude that has converged with the knowledge that there is data about their body’s functions and their health, and they want to see it. Hugo Campos is a famous example. An implanted cardioverter-defibrillator (ICD) manages his life-threatening heart condition, something he is happy about, but he does not want to wait around passively for the next time it shocks him. He wants to know how to manage his life, but the device’s maker, Medtronic, will not give him the data that comes out of his own heart. Thus, they keep the information and block Hugo from managing his own life. The extraordinary health researcher/anthropologist/thinker Susannah Fox has just capped off the ten years I have known her with a new post about Hugo and their related issues, “Why should anyone care about health data interoperability?” [34]

However, the most spectacular example of empowered patients is the OpenAPS open-source pancreas system. These people invented something that works better than a healthy pancreas at managing their blood sugar, with no doctors or scientists or device makers involved [35,36]. Their original hashtag is a perfect expression of liberation, #WeAreNotWaiting, which, when merged with technology, has produced a new, patient-created way to manage a life-threatening condition [37,38]. The most hopeful development on the horizon, to me, is that Sloan School of Management professor Eric von Hippel has begun applying his thinking on “Free Innovation” (a book about consumers modifying products or creating inventions) to health care. In February, he, his daughter, and others authored, “When Patients Become Innovators” in the Sloan Management Review. The featured patients were Dana Lewis and the OpenAPS group, and Sean Ahrens of Crohnology, which is a community of Crohn’s Disease patients.

## Open Access Embodies All the Issues of the Past Decade

We have learned that if EMR information is held only by third parties, then we the patients will depend on them for everything. We have learned that getting our hands on EMR data, such as through OpenNotes, enables real change and contribution. Similarly, if access to medical journals is available only to professionals (health care providers and researchers), then we, the patients, for whose benefit they work, are oppressed, whether that is intentional or not. However, we have seen, through JMIR and others, that Open Access journals can accelerate the dissemination of new knowledge, which increases the aggregate potential of what health care can achieve.

We must revolt against the limits that constrain progress. We must speak up, say what is important to us, and argue ceaselessly for the change we want in the world. Often, we may only shift from that dependent role when some trigger provides motivation, but this is not a shift someone can perform while their boat is metaphorically sinking. Empowerment through information can be revolutionary, but it is not instantaneous; when we acquire a new asset, we must learn to use it. Let us educate each other before trouble strikes, so that when it does, we have the best odds of the kind of success other highly engaged patients and I have achieved: to help health care achieve its potential.

## Abbreviations

CIO: chief information officer
EMR: electronic medical record
e-patient: electronic patient
FHIR: Fast Health care Interoperability Resources
ICD: implanted cardioverter-defibrillator
JMIR: Journal of Medical Internet Research
